# Breathlessness and sexual activity in older adults: the Australian Longitudinal Study of Ageing

**DOI:** 10.1038/s41533-018-0090-x

**Published:** 2018-06-22

**Authors:** Magnus Ekström, Miriam J. Johnson, Bridget Taylor, Mary Luszcz, Pia Wohland, Diana H. Ferreira, David C. Currow

**Affiliations:** 1 Department of Respiratory Medicine and Allergology, Institution for Clinical Sciences, Lund University, Lund, Sweden; 2 IMPACCT, Faculty of Health, University of Technology Sydney, Ultimo, NSW Australia; 3 Wolfson Palliative Care Research Centre, Hull York Medical School, University of Hull, Hull, UK; 4 Sobell House Hospice, Oxford University Hospitals NHS Foundation Trust, Oxford, UK; 5 School of Psychology, and Centre for Ageing Studies, Flinders University, Adelaide, SA Australia; 6 Institute for Clinical and Applied Health Research, Hull York Medical School, University of Hull, Hull, UK; 7 Discipline, Palliative and Supportive Services, Flinders University, Adelaide, SA Australia

## Abstract

Sexual activity is important to older adults (65 + ). Breathlessness affects about 25% of older adults but impact on sexual activity is unknown. We evaluated the relationships between breathlessness and sexual inactivity and self-reported health among older community-dwelling adults in the Australian Longitudinal Study of Ageing. Associations between self-reported breathlessness (hurrying on level ground or walking up a slight hill) at baseline, self-reported sexual activity, overall health and health compared to people of the same age were explored using logistic regression at baseline and 2 years, adjusted for potential confounders (age, sex, marital status, smoking status and co-morbidities). Of 798 participants (mean age 76.4 years [SD, 5.8] 65 to 103; 53% men, 73% married), 688 (86.2%) had 2-year follow-up data. People with breathlessness had higher prevalence and duration of sexual inactivity (77.7% vs. 65.6%; *p* < 0.001; 12 [IQR, 5–17] vs. 9.5 [IQR, 5−16] years; *p* = 0.043). Breathlessness was associated with more sexual inactivity, (adjusted OR 1.75; [95% CI] 1.24−2.45), worse health (adjusted OR 2.02; 1.53−2.67) and worse health compared to peers (adjusted OR 1.72; 1.25−2.38). Baseline breathlessness did not predict more sexual inactivity at 2 years. In conclusion, breathlessness contributes to sexual inactivity and worse perceived health in older adults, which calls for improved assessment and management.

## Breathlessness: Links with levels of sexual activity in over 65s

Treating breathlessness in older people could help improve well-being and prolong their sex lives. Chronic breathlessness in older age has psychological effects on perceived physical health, and can cause depression and anxiety, all of which can impact on levels of sexual activity. However, little data is available on the links between self-reported breathlessness and sexual activity in over 65s. Magnus Ekstrom at the University of Technology Sydney and co-workers analysed data from 688 people that participated in the Australian Longitudinal Study of Ageing to explore breathlessness, perceived health and sexual activity. People suffering breathlessness were more likely to have longer periods without sexual activity compared to those without the condition. They also reported their overall health to be poorer. Further in-depth research is required into the impact of chronic breathlessness on sexual activity with age.

## Introduction

There is a paucity of information on sexual activity in breathlessness to guide clinicians supporting older adults. Research has shown that sexual activity remains important to older adults,^1–4^ including those with profound disability.^5–7^ Sexual inactivity is associated with older age, living alone and poor overall health.^8^ British interviewees aged 50–92 years described their own or their partner’s health problems as the biggest barrier to sexual activity.^2^ Sadly, although society is developing more permissive attitudes, there remains a stigma surrounding sexual activity in older adults.^9^ It is, therefore, imperative that study is afforded to this area of health, which has an important impact on quality of life.

Breathlessness is common and affects between 17% to over a third of older adults (70 + ).^10^ Breathlessness is a predictor of shorter survival;^11^ increases in prevalence in the months prior to death;^11^ and has a significant negative impact on function in older adults.^10^ It limits mobility, leisure activities and the capacity to perform basic activities of daily living^10^ with physical and mental components of quality of life decreasing as breathlessness worsens.^12^ Although ill-health is identified as a reason for reduced sexual activity,^2,13^ little is known about the impact of breathlessness on older people’s sexual activity and self-reported health.^2^ As breathlessness has evidence-based interventions for benefit, this could represent a therapeutic target in this area for affected individuals.

The aim of this article is to evaluate the association of breathlessness with sexual inactivity and self-reported health among older adults (65 + ) living in the community using data from the Australian Longitudinal Study of Ageing (ALSA). The prevalence and characteristics of breathlessness, sexual inactivity and self-reported health is described and the relationship between breathlessness, sexual inactivity and self-reported health at baseline and after 2 years explored.

## Results

### Participants

Of the 798 participants that met the eligibility criteria, the mean age was 76.4 years (SD, 5.8); 53.4% were men, and 72.7% were married (Table 1). Characteristics were similar for people included and excluded in the analysis, both at baseline and follow-up (Supplementary Table S1 of the online supplement).

**Table 1:** Characteristics of 798 older people living in the community

Characteristic at baseline	Without breathlessness *N* = 421 (52.8%)	With breathlessness *N* = 377 (47.2%)	*p*-value
Age, years	76.0 ± 5.8	76.9 ± 5.8	0.029
Men	227 (53.9)	199 (52.8)	0.75
Living alone	105 (24.9)	103 (27.3)	0.44
Married	309 (73.4)	271 (71.9)	0.63
Home type			< 0.001
House	333 (79.1)	227 (60.2)	
Home unit or flat	77 (18.3)	133 (35.3)	
Other	9 (2.2)	15 (4.0)	
Not reported	2 (0.5)	2 (0.5)	
Body mass index, kg/m^2^	25.4 ± 3.8	26.3 ± 4.0	0.001
Number of co-morbidities	4.8 ± 2.6	6.2 ± 3.0	< 0.001
Asthma	11 (2.6)	45 (11.9)	< 0.001
Breast cancer	9 (2.1)	6 (1.6)	0.57
Chronic bronchitis	29 (6.9)	61 (16.2)	< 0.001
CES-D score	8.2 ± 15.4	10.6 ± 14.6	0.024
Depression (CES-D ≥ 16)	43 (10.2)	69 (18.3)	0.001
Diabetes mellitus	33 (7.8)	31 (8.2)	0.84
Gynaecological or urological condition	91 (21.6)	104 (27.6)	0.050
Heart disease	66 (15.7)	136 (36.1)	< 0.001
Hypertension	131 (31.1)	130 (34.5)	0.31
Joint pain^a^	152 (36.1)	181 (48.0)	< 0.001

Data presented as frequency (percentage) or mean ± standard deviation

*CES-D* Centre for Epidemiologic Studies Depression Scale, *SD* standard deviation

^a^Joint pain was assessed using the question ‘Have you had joint pains for most days during the last months? (yes/no)’

Compared to people without breathlessness, people with breathlessness had more co-morbidities (asthma, chronic bronchitis, heart disease, gynaecological or urological conditions, joint pain and depression) (Table 1). People with or without breathlessness were similar in terms of age, body mass index (BMI), marital status, proportion of people living alone, prevalence of diabetes mellitus, breast cancer and hypertension (Table 1).

Follow-up data at 2-years were available for 688 (86.2%) participants. Continuation rates were similar between people with and without breathlessness at baseline (84.9% vs. 87.4%; *p* = 0.30).

### Sexual inactivity

At baseline, 569 (71.3%) participants reported sexual inactivity with a median duration of 11 (IQR, 5−17) years. Sexual inactivity was more common (Table 2) and of longer duration in people with breathlessness than people without breathlessness (prevalence 77.7% vs. 65.6%; *p* < 0.001; duration 12 [IQR, 5−17] years vs. 9.5 [IQR, 5−16] years; *p* = 0.043).

**Table 2:** Breathlessness and sexual inactivity and self-rated health

	Without breathlessness at baseline *N* = 421 (52.8%)		With breathlessness at baseline *N* = 377 (47.2%)		*p*-value^a^
Baseline	*N* observations		*N* observations		
Sexually inactive	421	276 (65.6)	377	293 (77.7)	< 0.001
Self-reported cause of sexual inactivity	266		285		0.81
Health reasons		80 (30.1)		91 (31.9)	
Loss of interest		60 (22.6)		70 (24.6)	
Loss of partner		58 (21.8)		49 (17.2)	
Partner’s loss of interest		48 (18.1)		49 (17.2)	
Self-rated health (higher is worse)	421	2.4 ± 0.9	377	3.1 ± 1.1	< 0.001
Median (IQR)	421	2 (2−3)	377	3 (2−4)	< 0.001
Self-reported health compared to perception of others’ health of the same age	415	1.3 ± 0.5	369	1.5 ± 0.6	< 0.001
Better		300 (71.3)		201 (53.3)	
Same		102 (24.2)		143 (37.9)	
Worse		13 (3.1)		25 (6.6)	
Not reported		6 (1.4)		8 (2.1)	
Follow-up at 2 years	*N* = 368 (87.4%)		*N* = 320 (84.9%)		0.30
Sexually inactive	268	195 (72.8)	243	185 (76.1)	0.38
Change from baseline in sexual inactivity	268	0.07 ± 0.38	243	0 ± 0.33	0.034
-1 (from inactive to active)		11 (4.1)		13 (5.4)	
0 (no change)		228 (85.0)		217 (89.3)	
1 (from active to inactive)		29 (10.8)		13 (5.4)	
Median (IQR)		0 (0−0)		0 (0−0)	0.033
Self-rated health (higher is worse)	364	2.6 ± 1.0	315	3.1 ± 1.0	< 0.001
Median (IQR)		2 (2−3)		3 (2−4)	< 0.001
Change from baseline in self-reported health (higher is worse)	364	0.16 ± 0.9	315	0.08 ± 1.0	0.28
-2		15 (4.1)		15 (4.8)	
-1		58 (15.9)		69 (21.9)	
0 (no change)		176 (48.4)		129 (41.0)	
1		83 (22.8)		79 (25.1)	
2		32 (8.8)		23 (7.3)	
Median (IQR)		0 (0−1)		0 (-1−1)	0.31

Data presented as frequency (percentage) or mean ± standard deviation

*CES-D* Centre for Epidemiologic Studies Depression Scale, *IQR* interquartile range, *SD* standard deviation

^a^Continuous variables were compared using *t*-test, dichotomous using chi-2 test and ordinal scales using Wilcoxon rank-sum test

**Fig. 1:**
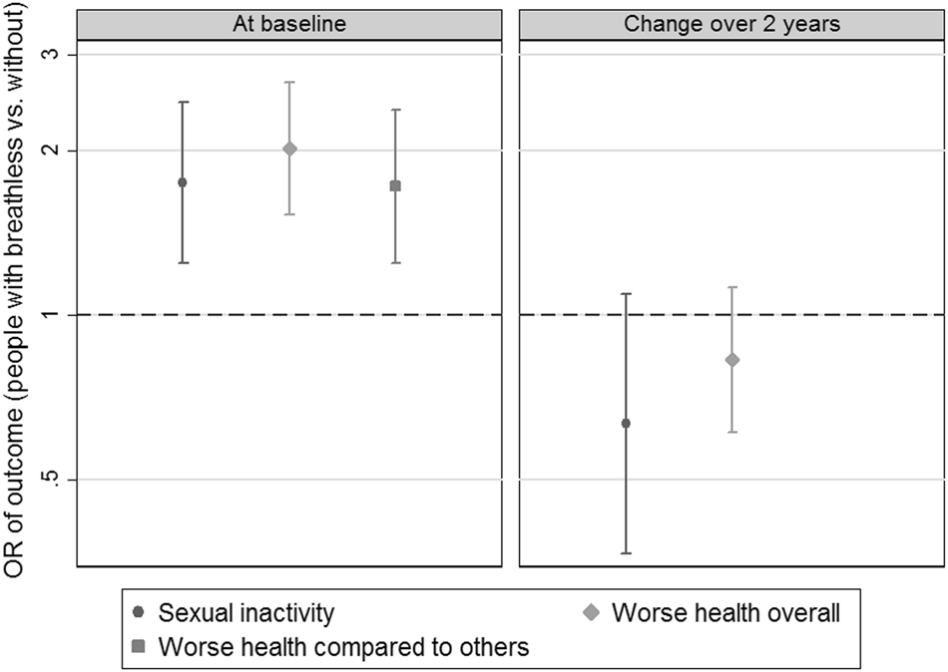
Associations between breathlessness at baseline and sexual inactivity, worse self-perceived health overall and compared with other people of the same age. Outcomes were analysed at baseline and as change from baseline at 2 years. Estimates are expressed as odds ratios (OR) with 95% confidence intervals, comparing people with breathlessness to people without. The estimates for sexual inactivity were adjusted for age, sex, living alone, smoking status, history of gynaecological or urological conditions, and joint pain. Estimates for self-reported health are adjusted for age, sex, living alone, smoking status, number of recorded morbidities, presence of diabetes mellitus, gynaecological or urological conditions (including incontinence), heart disease, hypertension, respiratory problem and joint pain

Breathlessness was associated with higher prevalence of sexual inactivity both in crude analysis (OR 1.83; 95% CI, 1.34 to 2.51) and in multivariable analysis adjusting for confounders (OR 1.75; 95% CI, 1.24 to 2.45) (Fig. 1). Other independent predictors of sexual inactivity were older age, being female, living alone, current smoking, a gynaecological or urological condition, and joint pains (Table 3)). Self-reported causes of sexual inactivity were similar between people with and without breathlessness (Table 2).

**Table 3:** Factors independently associated with sexual inactivity in older people in the community

Factor	Odds ratio	95% Confidence interval
Age (per 1 year)	1.10	1.06–1.14
Female	2.21	1.49–3.29
Breathlessness	1.75	1.24–2.45
Living alone	2.79	1.73–4.49
Never-smoked	1.0	
Ex-smoker	0.78	0.54–1.13
Smoker	2.35	1.10–5.02
Gynaecological or urological condition	1.47	0.98–2.22
Joint pains	1.35	0.96–1.89

Adjusted associations with sexual inactivity using multivariable logistic regression. Each estimate is adjusted for all other factors in the model

Sexual inactivity was associated with significantly worse self-rated health overall (OR 1.42; 95% CI, 1.05 to 1.92) and worse perceived to perceptions of others’ health the same age (OR 1.60; 95% CI, 1.11 to 2.31). Longitudinally, baseline breathlessness was not associated with sexual inactivity after 2 years, OR 1.12 (95% CI, 0.74 to 1.71), nor with increased risk of becoming sexually inactive (Fig. 1).

### Self-rated health

Self-reported health at baseline was worse (higher scores) in people with breathlessness both overall (3.1 vs. 2.4; *p* < 0.001) and compared with the perceived health of others of similar age (Table 2). Breathlessness was associated with worse health overall both in crude (OR 3.13; 95% CI, 2.41−4.07) and adjusted analysis (OR 2.02; 95% CI, 1.53−2.67) (Fig. 1). This relationship was also seen for perceived health compared to others the same age (crude OR 2.18 [95% CI, 1.63−2.93]; adjusted OR 1.72 [95% CI, 1.25−2.38]) (Fig. 1). In longitudinal analysis, breathlessness at baseline was associated with worse self-perceived health 2 years later (OR 1.72; 95% CI, 1.28−2.33) but not with worsening health over time (Fig. 1).

### Sensitivity analyses

There were no evidence that the association of breathlessness differed between men and women for any of the outcomes (*p* > 0.30 for all interaction terms with sex). In a sensitivity analysis among people not living alone (*N* = 590), all findings were robust; adjusted associations between breathlessness and sexual inactivity, OR 1.67 (95% CI, 1.16−2.40); self-rated health, OR 1.92 (95% CI, 1.39−2.66); self-rated health compared to others same age, OR 2.00 (95% CI, 1.37−2.90).

## Discussion

This is the first study showing a positive association between breathlessness and both prevalence and longer duration of sexual inactivity in older people However, baseline breathlessness did not predict a change in sexual inactivity at 2 years’ follow-up. Consistent with the findings of earlier research,^2^ increasing age, living alone and poorer overall health were also associated independently with sexual inactivity. Participants’ reported reasons for sexual inactivity were loss of partner, loss of interest and one’s own or one’s partner’s health. This large, prospectively collected data set contributes new associations between sexual inactivity and breathlessness.

Epidemiological studies of older men report direct associations between impotence and physical causes such as medication for hypertension, heart disease and diabetes,^14^ and confirm the importance of maintaining any level of erectile stiffness.^1^ However, apart from any physical causes, the dynamics of couples’ emotional and sexual relationships will also play a part. A person’s physical condition, psychological well-being and relationship with their partner are cornerstones of sexuality; together providing a conceptual framework for the complex impact of chronic diseases and increasing frailty on sexual function and well-being.^15^ Chronic breathlessness fits this conceptual framework: it is associated with physical disability,^16^ decreased psychological well-being,^12^ and increased depression and anxiety.^17^ Breathlessness can impact significantly on intimate relationships once a spouse or partner becomes the main carer with additional burden and responsibility.^18^

Earlier UK research found that older adults (aged 50–92 years) who rated sexual activity of low or no importance commonly expressed the expectation that sexual interest and sexual activity naturally declined with age.^2^ When there were barriers to sexual activity, the role of sex was reprioritized; affording greater importance to maintaining physical intimacy through touch and hugging. As the ALSA survey posed a single question about sexual activity, we are unable to establish whether older people’s interpretations of ‘sexual activity’ were limited to penetrative sexual intercourse, or whether all sexual activity was considered. More permissive attitudes to a variety of sexual practices might influence these findings if the study were repeated today.

### Strengths and limitations

This is the first study of sexual inactivity and self-perceived health in relation to breathlessness in older adults. Strengths of the study include the population-based cohort of the relevant age group with prospectively collected, longitudinal data. Analyses were controlled for a number of relevant confounders and evaluated associations at baseline and follow-up, as well as change in sexual activity and self-perceived health over time.

Data on breathlessness were unfortunately only available at baseline. It is not known if breathlessness resolved in some people or developed over time in those without breathlessness at baseline; that is, the trend of increased sexual activity, albeit non-statistically significant, observed in the breathlessness group could be due to regression towards the mean.

Although individuals’ own perceptions of the reason for sexual inactivity are important, they only provide a partial picture. Data on sexual activity were unavailable for 54% of participants. As people with sexual problems, or who are sexually inactive, may be less likely to provide data, sexual activity in this age group may be over-estimated and the impact of breathlessness under-estimated. However, the characteristics of included and excluded people were similar. Moreover, findings were similar when excluding people living alone, supporting the validity of the findings.

This study is based in Australia, which may limit the generalisability of the data. However, the importance of the area of study is mirrored in the literature from different cultures and less well-resourced nations.^1,4,13^

### Implications for research

Sexual well-being is a subjective construct, which is poorly correlated with levels of sexual activity.^15,19^ The impact of breathlessness on both sexual function and sexual well-being requires further research. This should specify what is meant by sexual activity; limiting this to penetrative sex overlooks a repertoire of sexual practices that may, in themselves, be fulfilling or less impeded by breathlessness, ageing, or widowhood. Valid and reliable standardised tools should be used that are applicable for differing genders and sexual orientations and include all important domains of sexual desire, activity and satisfaction.^20^ Likewise, breathlessness is a multidimensional symptom that affects people differently depending on its severity and chronicity. Future research should address the specific impact of chronic breathlessness on sexuality by mMRC score, which identifies people that have limited and very limited activity due to breathlessness and those that are housebound.

Longitudinal data on breathlessness and sexual activity would help explore the potential of breathlessness as a therapeutic target for people who wanted to become sexually active again. Systematic inclusion of sexual activity in breathlessness clinical study outcomes would be useful. Qualitative research in this area could provide valuable insights about the extent to which couples modify and adapt their sexual behaviour in response to chronic breathlessness.

### Implications for clinical practice

Health care professionals are often reluctant to discuss sexual issues proactively with patients, especially with older adults.^21–24^ Embarrassment, lack of knowledge and low confidence are important limiting factors,^21–24^ while stereotypical views about an asexual older age underpin a belief that sexual health is not a ‘legitimate’ topic for discussion.^21^ This is compounded by the reticence of older adults to initiate discussion about sexual concerns due to lack of knowledge, shame, fear or embarrassment.^25^ However, concerns or fears about the effect of sexual activity on breathlessness, are likely to adversely affect sexual and emotional intimacy. If introduced in initial assessments as part of routine enquiry, sexual activity and intimacy can more easily be revisited ^26^ and sensitive discussion and management of these matters facilitated.^27^ Resources to facilitate this discussion are available.^28^

## Conclusion

This large, prospective study of community-dwelling older adults shows that breathlessness is associated with sexually inactivity and impaired self-reported health, having controlled for other key factors. Further work should evaluate the effect of optimal symptomatic management of chronic breathlessness on sexual activity over time. This would require a much larger cohort of older people to be followed prospectively while specifically evaluting what level of sexual activity was sought by participants. These findings convey the importance of clinicians systematically inquiring about sexual activity in clinical practice when caring for older adults’. for whom maintaining or re-engaging in sexual activity is an important goal of care.

## Material and methods

### Design and population

This study analysed data from ALSA, a longitudinal population-based study established in 1992 of 2087 older adults aged 65 to 103 years living at home or in residential care in metropolitan Adelaide, South Australia, as detailed elsewhere.^29^

Inclusion criterion for the present analysis was people living independently in the community (*n* = 1961 partcipants). Exclusion criteria were missing data on any of the three target dependent variables (breathlessness, sexual activity and self-perceived health) (*n* = 1060) and inability or unwillingness to walk uphill as the question on breathlessness was in the context of walking (*n* = 103).

Data on 798 participants remained for analysis. The associations between breathlessness, sexual inactivity and self-reported health were analysed longitudinally, at baseline (1992) and at the next follow-up point 2 years later (October–December 1994).^29^ Later follow-up time points were not analysed due to higher rates of missing data on sexual activity.

### Data collection

#### Baseline covariates

ALSA collected data during a home visit at baseline including participant-completed questionnaires on demographics; living conditions; marital status coded as co-habiting (married or de facto) or living alone (separated, divorced, widowed or never married); smoking status (never, former and current), history of morbidities including respiratory problems (asthma or chronic bronchitis), diabetes mellitus, heart disease (heart attack or other heart condition), hypertension, breast cancer, prostate cancer or prostate problem, gynaecological cancer or condition and urological conditions (including incontinence). For this analysis, prostate and other urogenital conditions were merged into the category ‘gynaecological or urological conditions’ due to small numbers. Risk of depression at baseline was assessed using the Centre for Epidemiologic Studies Depression Scale (CES-D).^30^ CES-D is a 20-item measure of experienced symptoms over the past week associated with depression, such as restless sleep, poor appetite, and feeling lonely. Scores ranged from 0 to 60, with higher scores indicating greater depressive symptoms.^30^ Depression was categorised as a CES-D score ≥ 16.^30^ Height and weight were measured at baseline and used to calculate BMI as weight(kg)/height(m)^2^.

#### Breathlessness

Breathlessness was assessed at baseline with the question ‘Are you troubled by shortness of breath when hurrying on level ground or walking up a slight hill?’ (yes/no). This level of breathlessness corresponds to a modified Medical Research Council (mMRC) breathlessness score ≥ 1.^31^

#### Sexual activity

Self-reported sexual activity was assessed at a visit scheduled about 2 weeks after the initial baseline visit and at follow-up using the question ‘Are you still sexually active?’ (yes/no). The numbers of years of sexual inactivity and self-reported reasons (health reasons, loss of interest, loss of partner, or partner’s loss of interest) were also recorded.

#### Self-reported health

Self-rated overall health (‘How would you rate your overall health at the present time?’) was assessed at baseline and follow-up on a 5-point ordinal scale (1 = excellent; 2 = very good; 3 = good; 4 = fair; 5 = poor). Data on health were compared with the participant’s perceived health compared to others of the same age (‘Would you say that your health is better, about the same, or worse than most people your age?’; coded as 1 = better; 2 = same; 3 = worse).

### Ethical considerations

Ethical approval was provided by the Flinders Medical Centre, Committee on Clinical Investigation (ID: Research Application 002-100andrews) at the instigation of the ALSA and renewed annually. Written, informed consent was obtained from all participants before each study wave.

### Statistical methods

Characteristics at baseline were tabulated using mean with standard deviation (SD) or median with interquartile range (IQR) for continuous variables with normal and non-normal distribution, respectively, and frequency (percentage) for categorical variables. Means were compared using *t*-test and medians using Wilcoxon rank-sum test. Characteristics were compared between people included and excluded in the analysis at baseline and follow-up.

Associations of interest were between (1) breathlessness, sexual inactivity and self-reported health at baseline; (2) sexual inactivity and self-reported health at baseline; and (3) breathlessness at baseline and change in sexual activity and change in self-reported health between baseline and follow-up at 2 years. Potential confounders (Table 1) included in each analysis were selected based on plausible biological rationale and evidence from the literature.^14^ Each factor was added separately and dropped if there was no substantial change in the estimate of interest. Thirdly, all factors were evaluated simultaneously to check the robustness of the final estimates.

In the final multivariable analysis, associations with sexual inactivity were analysed using logistic regression adjusted for age, sex, living alone, smoking status, history of gynaecological or urological conditions, and joint pain. Self-reported health, overall health compared to others of the same age, and changes from baseline in sexual inactivity and overall health were analysed using ordinal logistic regression, adjusted for age, sex, living alone, smoking status, number of recorded morbidities, the presence of diabetes mellitus, gynaecological or urological conditions (including incontinence), heart disease, hypertension, respiratory problem, and joint pain. Whether the associations for breathlessness differed between men and women were evaluated by including interaction terms between breathlessness and sex in each fully adjusted model. As depression could be both a cause and a consequence of breathlessness, a sensitivity analysis adjusting for depression was performed. Associations were expressed as odds ratios (OR) with 95% confidence intervals (CI). Statistical significance was defined as a two-sided *p*-value < 0.05. Statistical analyses were performed with Stata version 14.2 (StataCorp LP; College Station, TX, USA).

### Data availability

The data that support the findings of this study are available from the corresponding author upon reasonable request.

## Electronic supplementary material

Table S1
