# Supplementary material for: Breathlessness and sexual activity in older adults: the Australian Longitudinal Study of Ageing
Source: NPJ Prim Care Respir Med. 2018 Jun 22;28:20. doi: 10.1038/s41533-018-0090-x (PMC6015074; doi:10.1038/s41533-018-0090-x)
Supplement: Supplementary file 1 — Table S1 [file 41533_2018_90_MOESM1_ESM.docx]

**Online supplement**

**Breathlessness and Sexual Activity in Older People: The Australian Longitudinal Study of Aging**

**Table S1. Characteristics of people who were included and excluded in the analysis at baseline and follow-up**

|  |  |  |  |  |  |  |  |  |  |  |  |  |
| --- | --- | --- | --- | --- | --- | --- | --- | --- | --- | --- | --- | --- |
|  |  | *All in ALSA^*^ N=1961* | | *Included at baseline N=798* | | *Excluded at baseline*  *N=1163* | | *Included at follow-up N=688* | | *Excluded at follow-up N=110* | |  |
|  |  | **Mean** | **SD** | **Mean** | **SD** | **Mean** | **SD** | **Mean** | **SD** | **Mean** | **SD** |  |
|  | Age (years) | 77.6 | 6.4 | 76.4 | 5.8 | 78.5 | 6.6 | 76 | 5.7 | 78.6 | 6.3 |  |
|  | Weight (kg) | 68.5 | 12.8 | 69.1 | 12.4 | 68.1 | 13.1 | 69.3 | 12.3 | 67.6 | 12.8 |  |
|  | Body mass index, kg/m2 | 25.9 | 4 | 25.9 | 3.9 | 26 | 4.1 | 25.9 | 3.9 | 25.4 | 3.9 |  |
|  | CES-D score | 7.9 | 7.2 | 7.3 | 6.6 | 8.4 | 7.5 | 7 | 6.4 | 9 | 7.9 |  |
|  | Total years smoking | 55.4 | 11.8 | 52.4 | 9.9 | 57.1 | 12.4 | 52.6 | 10.5 | 51.6 | 7.7 |  |
|  | Cigarettes per day before quitting | 19.6 | 16.4 | 18.7 | 16.1 | 20.3 | 16.5 | 18.6 | 16.4 | 19.4 | 14.6 |  |
|  |  |  |  |  |  |  |  |  |  |  |  |  |
|  |  | *Count* | *%* | *Count* | *%* | *Count* | *%* | *Count* | *%* | *Count* | *%* |  |
|  | Men | 1003 | 51.1 | 426 | 53.4 | 577 | 49.6 | 357 | 51.9 | 69 | 62.7 |  |
|  | Women | 958 | 48.9 | 372 | 46.6 | 586 | 50.4 | 331 | 48.1 | 41 | 37.3 |  |
|  | Married | 1336 | 68.1 | 580 | 72.7 | 756 | 65 | 505 | 73.4 | 75 | 68.2 |  |
|  | Born in Australia | 1330 | 67.8 | 570 | 71.4 | 760 | 65.3 | 494 | 71.8 | 76 | 69.1 |  |
|  | Living alone | 1436 | 73.2 | 590 | 73.9 | 846 | 72.7 | 514 | 74.7 | 76 | 69.1 |  |
|  | Home Type |  |  |  |  |  |  |  |  |  |  |  |
|  | Home Unit or flat | 521 | 26.6 | 210 | 26.3 | 311 | 26.7 | 179 | 26 | 31 | 28.2 |  |
|  | House | 1381 | 70.4 | 560 | 70.2 | 821 | 70.6 | 487 | 70.8 | 73 | 66.4 |  |
|  | Other | 37 | 1.9 | 24 | 3.1 | 13 | 1.1 | 18 | 2.6 | 6 | 5.4 |  |
|  | Smoking status |  |  |  |  |  |  |  |  |  |  |  |
|  | Never smoked | 939 | 47.9 | 370 | 46.4 | 569 | 48.9 | 326 | 47.4 | 44 | 40 |  |
|  | Ex-smoker | 839 | 42.8 | 366 | 45.9 | 473 | 40.7 | 312 | 45.3 | 54 | 49.1 |  |
|  | Smoker | 173 | 8.8 | 62 | 7.8 | 111 | 9.5 | 50 | 7.3 | 12 | 10.9 |  |
|  | Diagnosed with chronic bronchitis | 227 | 11.6 | 90 | 11.3 | 137 | 11.8 | 75 | 10.9 | 15 | 13.6 |  |
|  | Currently suffers from chronic bronchitis | 134 | 6.8 | 60 | 7.5 | 74 | 6.4 | 48 | 7 | 12 | 10.9 |  |
|  | Diagnosed with diabetes | 169 | 8.6 | 64 | 8 | 105 | 9 | 50 | 7.3 | 14 | 12.7 |  |
|  | Currently suffers from diabetes | 131 | 6.7 | 44 | 5.5 | 87 | 7.5 | 35 | 5.1 | 9 | 8.2 |  |
|  | Diagnosed with heart attack | 243 | 12.4 | 89 | 11.2 | 154 | 13.2 | 78 | 11.3 | 11 | 10 |  |
|  | Currently suffers from heart attack | 81 | 4.1 | 19 | 2.4 | 62 | 5.3 | 18 | 2.6 | 1 | 0.9 |  |
|  | Diagnosed with heart condition | 369 | 18.8 | 141 | 17.7 | 228 | 19.6 | 116 | 16.9 | 25 | 22.7 |  |
|  | Currently suffers from heart condition | 272 | 13.9 | 97 | 12.2 | 175 | 15 | 77 | 11.2 | 20 | 18.2 |  |
|  | Diagnosed with hypertension | 631 | 32.2 | 261 | 32.7 | 370 | 31.8 | 227 | 33 | 34 | 30.9 |  |
|  | Currently suffers from hypertension | 480 | 24.5 | 197 | 24.7 | 283 | 24.3 | 172 | 25 | 25 | 22.7 |  |
|  | Gynaecological cancer | 33 | 1.7 | 10 | 1.3 | 23 | 2 | 10 | 1.5 | 3 | 2.7 |  |
|  | Breast Cancer | 36 | 1.8 | 15 | 1.9 | 21 | 1.8 | 12 | 1.7 | 1 | 0.9 |  |
|  | Prostate cancer | 31 | 1.6 | 11 | 1.4 | 20 | 1.7 | 10 | 1.5 | 0 | 0 |  |
|  |  |  |  |  |  |  |  |  |  |  |  |  |

* All living in community. *Abbreviations:* ALSA = Australian Longitudinal Study of Ageing; SD = standard deviation.
